# Engineering strategies and optimized delivery of exosomes for theranostic application in nerve tissue

**DOI:** 10.7150/thno.84971

**Published:** 2023-07-24

**Authors:** Qicheng Li, Xiaoyang Fu, Yuhui Kou, Na Han

**Affiliations:** 1 Department of Trauma and Orthopedics, Peking University People's Hospital, Beijing 100000, China.; 2 Key Laboratory of Trauma and Neural Regeneration (Peking University), Ministry of Education, Beijing 100000, China.; 3 National Center for Trauma Medicine, Beijing 100000, China.; 4 Department of Central Laboratory and Institute of Clinical Molecular Biology, Peking University People's Hospital, Beijing 100000, China.

**Keywords:** biomaterials, engineering exosomes, exosomal miRNA, nerve injury, neuroimaging

## Abstract

Severe injuries or diseases affecting the peripheral and central nervous systems can result in impaired organ function and permanent paralysis. Conventional interventions, such as drug administration and cell-based therapy, exhibit limited effectiveness due to their inability to preserve post-implantation cell survival and impede the deterioration of adjacent tissues. Exosomes have recently emerged as powerful tools for tissue repair owing to their proteins and nucleic acids, as well as their unique phospholipid properties, which facilitate targeted delivery to recipient cells. Engineering exosomes, obtained by manipulating the parental cells or directly functionalizing exosomes, play critical roles in enhancing regenerative repair, reducing inflammation, and maintaining physiological homeostasis. Furthermore, exosomes have been shown to restore neurological function when used in combination with biomaterials. This paper primarily focuses on the engineering strategies and delivery routes of exosomes related to neural research and emphasizes the theranostic application of optimized exosomes in peripheral nerve, traumatic spinal cord, and brain injuries. Finally, the prospects of exosomes development and their combination with other approaches will be discussed to enhance our knowledge on their theranostic effectiveness in neurological diseases.

## Introduction

The central and peripheral nervous systems (CNS and PNS, respectively) are the most complex conduction systems in the human body. Severe injuries or diseases pose challenges to the recovery of the peripheral nerve, spinal cord, and brain because of substantial defects in the axons, vasculature, and neuronal deficits 1, 2. Conventional treatments involve the application of drugs or crosslinked polymers, including natural biomaterials (e.g., chitosan, collagen, and fibrin) or synthetic materials (e.g., PLGA and PLLA), which have been developed to facilitate the loading of the neural stem cells (NSCs), mesenchymal stem cells (MSCs), and growth factors for neural tissue repair 3, 4. However, the following challenges still persist in implementing these therapeutic strategies: (1) administration of high-dose hormones can result in serious side effects, and the presence of biological barriers prevents traditional drugs from reaching injured sites 5; (2) living cells loaded into biomaterials may not receive adequate blood supply during early transplantation, leading to decreased cell viability or cell death; (3) injectable biomaterials loaded with NSCs or MSCs require extensive clinical trials to prove their safety and efficacy in the human body; and (4) the short half-life and inadequate intracellular delivery of growth factors restrict their potential to promote the long-term growth of neighboring cells and axons.

**Figure 1:**
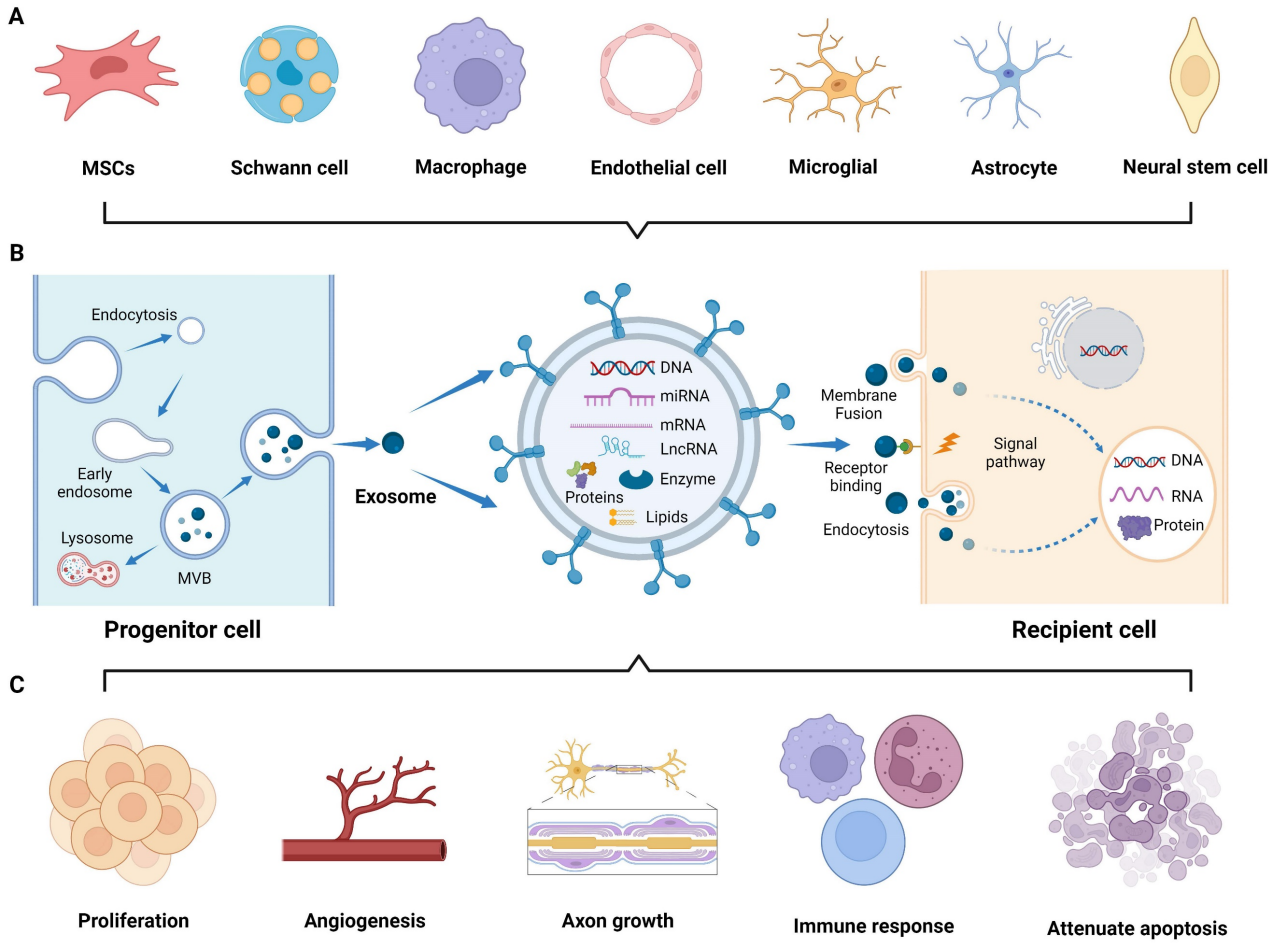
**Application of exosomes in nerve regeneration. (A)** Exosomes derived from various cells research. **(B)** Biogenesis and interaction of exosomes with recipient cells. **(C)** Application of exosomes to the sites of nerve injury, promoting cell proliferation, angiogenesis, and axon growth, modulating immune response, and attenuating cell apoptosis. Figures created with Biorender.com.

To overcome these limitations, researchers have devoted considerable effort to studying extracellular vesicles (EVs), including microvesicles, apoptotic bodies, and exosomes 6. In particular, exosomes, which can be biosynthesized and secreted by various cells (**Figure 1A**) 7-9, possess a nano-scale membrane structure (30-100 nm) that enables them to cross biological barriers. Moreover, upon entering the circulation, exosomes exhibit resistance to rapid degradation, and their specific molecules on the lipid bilayer shell allow them to target and fuse with distant cell membranes, thereby mediating intercellular communication. Additionally, the internal cavity of exosomes encapsulates a large number of signaling moieties, such as enzymes, transcription factors, and cytokines. Furthermore, the bilayer membrane structure exhibits hydrophilic and hydrophobic properties, endowing exosomes with cell-like carrier characteristics for tissue engineering purposes 10, 11. In the field of regenerative medicine, the utilization of biomaterials as exosomes carriers can achieve sustained and localized release of exosomes, which enhances the reparative effects mediated by biomaterials that are not loaded with exosomes 12. Extensive studies have been conducted on natural and engineered exosomes in animal models of neurological injuries, such as sciatic nerve, traumatic spinal cord, and brain injuries. In these studies, many similarities were observed in the therapeutic principles and delivery pathways, owing to the close structural relationship between the PNS and CNS.

Currently, there have been numerous reports on the preparation, transport mechanisms, and application of exosomes; however, existing literature lacks comprehensive reviews on the engineering strategies of exosomes and their application in nerve tissues. In this review, we will highlight the methods of functionalizing exosomes at the cellular and exosomal levels to enhance their efficacy in neuroregeneration and their delivery routes. We also discuss the application of exosomes in neuroimaging and their combination with nerve conduits, hydrogels, and 3D printed bioscaffold for treating peripheral nerve, traumatic spinal cord, and brain injuries, providing new insights into future developments in the field of regenerative medicine for nerve tissues.

## Biogenesis, interaction, and tissue source

In 1987, Johnstone et al*.* first described extracellular “exosomes” as a mechanism for shedding of specific membrane functions 13. Since then, the biogenesis of exosomes has been continuously reported, and it is now described as an endocytic pathway originating from cell membrane. Briefly, exosomes generation is usually divided into four steps: invagination, endosome formation, fusion, and secretion 14. The invagination of the cell membrane forms clathrin-coated vesicles by inward budding and then fuses together to form early endosomes in the cytoplasm. In the maturation process of endosomes, intraluminal vesicles (ILVs) begin to form through further invagination of the limiting endosomal membrane, a crucial step regulated by the endosomal sorting complex required for transport (ESCRT). Subsequently, the ILVs containing proteins, lipids, and nucleic acids develop into multivesicular bodies (MVBs). Some MVBs are transported to the lysosomes for degradation, while others fuse with the plasma membrane and release exosomes into the extracellular microenvironment (**Figure 1B**) 15. The released exosomes primarily interact with recipient cells through three routes 16: (1) complete endocytosis by the recipient cells, (2) direct membrane fusion between exosomes and recipient cells, and (3) direct interaction of transmembrane proteins on the exosomes with signaling molecules on the surface of the recipient cells, which play an important role in mediating gene expression and signaling pathways of the recipient cells by transferring the contents and biological signals carried by the exosomes (**Figure 1B**) 17.

In the field of neuroprotection research, numerous studies have demonstrated the role of exosomes originating from cells of the CNS, PNS, mesenchymal tissues, and other tissues in nerve regeneration. Exosomes derived from the CNS, including the brain and spinal cord, are released by the neurons 17, astrocytes 18, microglia 19, and oligodendrocytes 20. These exosomes specifically target brain and spinal cord diseases. The PNS is another valuable source of tissue-derived exosomes that have regenerative potential. Exosomes derived from the Schwann cells (SCs), the principal glial cells in the PNS, have been extensively studied 21. Furthermore, exosomes derived from other PNS components, such as macrophages, contribute to neural repair processes 22. Moreover, exosomes derived from various mesenchymal tissues, such as the bone marrow, adipose tissue, and umbilical cord, have shown promise in promoting nerve regeneration 23. Exosomes released by MSCs within these tissues carry diverse cargo molecules, including growth factors, cytokines, and membrane proteins, thereby promoting PNS and CNS repair and avoiding the risks associated with MSCs transplantation. Besides, exosomes derived from other tissues, such as the blood vessel and muscle, exert regenerative effects on injured nerves 8, 24. These tissue-derived exosomes enter the injured nerve area and participate in various regulatory events, including promoting axonal myelinization and angiogenesis; regulating immune responses; reducing cell apoptosis; and facilitating peripheral nerve, spinal cord, and brain injury repair (**Figure 1C**) 25-27. Nonetheless, the biofunction and yield of natural exosomes are inherently limited, underscoring the necessity to engineer both source cells and exosomes to enhance their theranostic potential in the nervous system.

## Pretreatment of progenitor cells to obtain engineering exosomes

**Figure 2:**
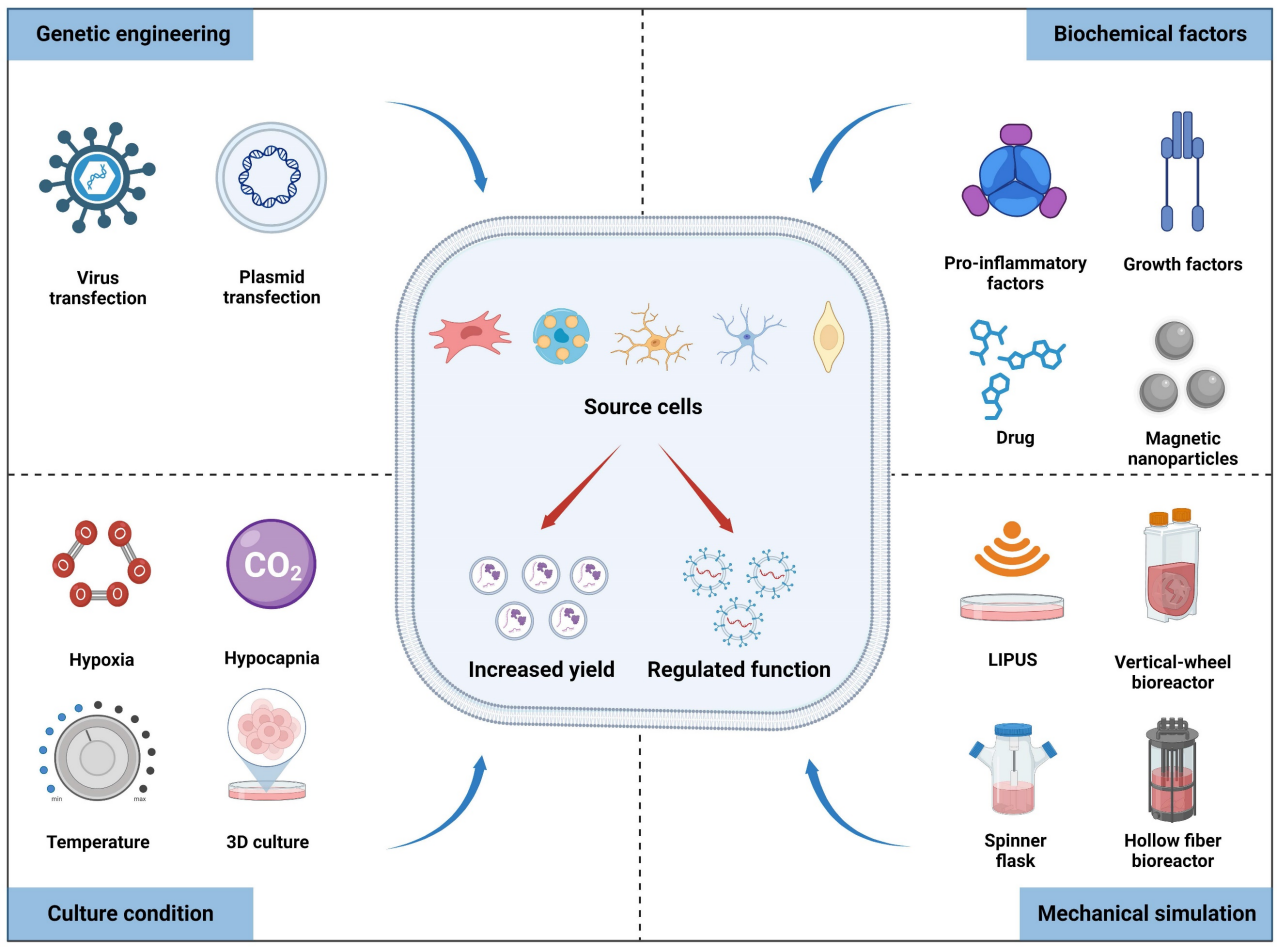
Multiple factors, including culture conditions, biochemical factors, mechanical stimulation, and genetic engineering, can affect the yield and biofunctions of exosomes derived from source cells. Figures created with Biorender.com.

Studies have shown that cell sources can regulate the contents of exosomes. For instance, the tissue source of MSCs determines the heterogeneity of proteins within secreted exosomes, leading to functional differences, as revealed by bioinformatics analysis 28. Furthermore, parental cells can promptly respond to environmental conditions and modulate the yield and bioactivity of nanovesicles, which is fundamental for achieving large-scale production and maintaining homeostasis. Therefore, various approaches at the cellular level have been employed to promote exosome production, and genetic engineering techniques can also be used to specialize them for enhancing tissue regeneration (**Figure 2**).

### Cell culture conditions

Cellular functions and phenotypes can be altered under different culture conditions, indirectly affecting the production and bioactivity of exosomes obtained from pretreated cells (**Table 1**). Previous studies have revealed that stem cells cultured under high-density conditions exhibit contact inhibition and enter a quiescent state 29. Conversely, lower-density cultivation can activate paracrine signaling pathways and promote exosomes secretion; therefore it is preferred to collect the supernatant for exosomes separation when cell confluence reaches 60% to 90% 30. Hypoxic preconditioning is a frequently used strategy to improve therapeutic outcomes prior to stem cell transplantation. Prior investigations have revealed that hypoxic preconditioning affects gene expression and proteome of the dental pulp stem cells cultured in a 3D system compared to normoxic conditions, resulting in the upregulation of the HIF-1α, VEGFA, and KDR mRNA, along with proteins involved in angiogenesis 31. Interestingly, exosomes produced from hypoxia-preconditioned human umbilical cord MSCs (hUCMSCs) group exhibited increased HIF-1α content compared to that of the normoxic group, thereby enhancing the pro-angiogenic effects in spinal cord injury *via* VEGF overexpression in the surrounding transplantation area 32. Consequently, hypoxic preconditioning may be an effective and promising method for exosomes engineering. Additionally, cells cultured in 3D culture system exhibit closer resemblance to *in vivo* state compared to traditional 2D culture methods, thereby augmenting the paracrine effects of MSCs and optimizing exosomes function 33. For example, UCMSCs cultured in a microcarrier-based 3D system resulted in a 20-fold increase in exosomes production compared to that of 2D culture. Additionally, when combined with tangential flow filtration (TFF), exosomes production was further amplified by seven times compared to 3D-derived exosomes alone. The delivery efficiency of siRNA to neurons using 3D-TFF-exosomes also increased by seven times 34. In conclusion, integrating diverse culture conditions with advanced isolation techniques can substantially boost the scale-up production and activity of exosomes.

### Generation of engineering exosomes *via* biochemical factors stimulation

The production and function of exosomes can be regulated by pretreating parental cells with biochemical factors, such as pro-inflammatory factors, magnetic nanoparticles, and growth factors (**Table 1**). Xu et al. treated MSCs with lipopolysaccharide (LPS), a typical pro-inflammatory factor 35. The generated exosomes significantly reduced the secretion of pro-inflammatory factors from LPS-stimulated RAW 264.7 cells, without affecting cell viability and apoptosis. The results of the western blot assay showed that AKT1 and AKT2 phosphorylation was activated through the NF-κB signaling pathway in M2 and M1 macrophages, which downregulated TNF-α, IL-6, and IL-1, thereby reducing inflammation response.

Harting et al. induced the secretion of anti-inflammatory exosomes from the bone mesenchymal stem cells (BMSCs) under IFN-γ and TNF-α stimulation to precisely target inflammation sites 36. Although the size distribution and surface markers of exosomes derived from inflammation-treated and naive MSCs were similar, differences were observed in their protein composition, cytokines, and RNA content, with further attenuation of the release of pro-inflammatory cytokines, partly due to the expression of COX2/PEG2 compared to naive MSCs-derived exosomes. The combined effects of exosomes and cytokines on immune regulation provide new insights into intercellular communication research. In addition to pro-inflammatory factors, Fe3O4 and iron oxide nanoparticles have been proven to regulate BMSCs-derived exosomes, exhibiting targeting and pro-angiogenic potential 37, 38. The engineered exosomes contained high levels of miR-21-5p, which promoted the expression of VEGF and HIF-1α in HUVECs by inhibiting SPRY2 and activating the PI3K/AKT and ERK1/2 signaling pathways, leading to cell proliferation and tubulogenesis. However, exosomes derived from high glucose-treated SCs further exacerbated the sciatic nerve impairment in diabetic db/db mice 39. In summary, progenitor cells preconditioned with appropriate biochemical factors can effectively improve the functional properties of exosomes and facilitate nerve repair.

### Improvement of exosomes yield *via* mechanical factors

Studies have found that mechanical stimulation, such as matrix stiffness, compressive, tensile, and shear stress, can modulate cellular characteristics 40, 41. For instance, an increase in fluid shear stresses promoted osteogenic differentiation and adipogenic dedifferentiation of MSCs *via* YAP expression 42. In recent years, many studies have displayed that mechanical cues alter the yield and biofunction of nanovesicles produced by progenitor cells. The shear stresses generated from dynamic systems, such as shake flasks, spinner flasks, roller bottles or bioreactors (mixing bubbles from spargers) affect cell phenotype and exosomes production 43. Yan et al. used a hollow-fiber bioreactor to culture UCMSCs and obtained exosomes with favorable biological functions and high yield (**Table 1**) 44. The hollow-fiber bioreactor possesses a large surface area that enables high-density cell seeding and facilitates the delivery of nutrients and oxygen to cells, while simultaneously diffusing and expelling cell metabolic waste through the hollow fibers. Compared to traditional 2D culture flasks, the bioreactor yielded 7.5-fold increase in the quantity of exosomes. Additionally, the 3D-derived exosomes exhibited significant advantages in promoting proliferation, migration, and matrix synthesis of recipient cells, which may be related to the activation of TGF-β1 and Smad2/3 signaling pathways. Similarly, Fuzeda et al. utilized a vertical-wheel bioreactor to culture MSCs from the bone marrow, adipose tissue, and umbilical cord seeded onto microcarriers (**Table 1**) 45. The shear stress exerted on cell membrane led to an increase in surface tensile strength and membrane contraction. Ultimately, the concentration and productivity of exosomes increased by 5.7-fold and 3-fold, respectively, compared with the static system. Apart from the aforementioned bioreactors, ultrasonic waves can generate shear stress on cell membranes. Ye et al. isolated exosomes derived from low-intensity pulsed ultrasound (LIPUS)-stimulated SCs to treat cavernous nerve crush injury 46. The results showed that LIPUS stimulation altered the miRNA expression of SCs-exosomes and enhanced nerve regeneration through the PI3K-Akt-FoxO signaling pathway (**Table 1**). These findings suggested that a scalable culture of exosomes-originating cells using mechanical stimulation can produce high-yield and functionally enhanced nanovesicles for the development of novel therapeutic products.

### Specific gene transfected into progenitor cells to alter exosomal content

Exosomes carry a diverse array of contents from donor cells and possess high heterogeneity, playing an important role in regulating the function of recipient cells. In 2007, Valadi et al. first discovered that mast cell-derived exosomes carried mRNA and miRNA, and the transferred exosomal mRNA can be translated into functional proteins in recipient cells 56. In addition, nucleic acid cargos loaded into exosomes also include DNA, ribosomal RNA (rRNA), long non-coding RNA (lncRNA), transfer RNA (tRNA), small nuclear RNA (snRNA), small nucleolar RNA, and p-element-induced wimpy testis (piwi)-interacting RNA 19. Among these, microRNA, the most abundant RNA species in exosomes, is a type of non-coding single-stranded RNA molecule encoded by endogenous genes and approximately 17-24 nucleotides in length; it participates in various biological processes in exosomes-mediated intercellular communication, such as angiogenesis, cell transport, cell apoptosis, and protein degradation 7, 16, 57.

Recent scientific reports on the utilization of miRNAs as gene carriers in exosomes for the diagnosis and treatment of neurological diseases have increased in number 58, 59. To enhance the regeneration of damaged axons, researchers have used genetic engineering techniques to deliver specific miRNAs, carried by viruses or plasmids, into recipient cells such as the MSCs, Schwann cells, and microglia, thereby increasing the production of engineered exosomes. Some studies have shown that MSCs overexpressing miRNAs are more effective at promoting functional recovery than normal MSCs. The overexpression of miR-133b in MSCs has been shown to improve the recovery of hindlimb function and promote axon regeneration in rats following compression spinal cord injury, achieved through the modulation of exosomal miR-133b, which activated ERK1/2, STATS, and CREB signaling pathway proteins 60. Exosomes and their miRNA cargos derived from MSCs also have immunoregulatory properties. Exosomes containing miR-125a derived from BMSCs reduced inflammatory responses and promoted M2 polarization. This study revealed the neuroprotective role of exosomal miR-125a through the negative regulation of IRF5 expression in SCI rats 61. Moreover, miRNA-loaded exosomes from different cells promote nerve repair by diminishing cell apoptosis, autophagy, and pyroptosis. Liang et al. discovered that the exosomal miR-499a-5p contributed to reducing neuronal apoptosis after OGD/R and confirmed its inhibitory effect on the JNK3/c-jun signaling pathway by targeting JNK3 26. Exosomes derived from ADMSCs, transported miRNA-26b to injured Schwann cells, leading to the suppression of cell autophagy, which was related to the downregulation of Kpna2. On the other hand, M2 microglia-exosomes enriched with miR-672-5p suppressed the AIM2/ASC/caspase-1 signaling pathway *via* inhibiting AIM2 activity, thereby negatively regulating neuronal pyroptosis and ultimately fostering the functional recovery of the spinal cord. Overall, increasing evidence of the involvement of exosomes and miRNAs in the treatment of nerve injuries highlights the potential of these molecules as therapeutic targets. The molecular mechanism of miRNA-containing exosomes in nerve repair is listed in **Table 2**.

## Direct functionalization of exosomes for nervous system

Although exosomes can be engineered at the cellular level to enhance their yield and function through the mechanism of protein production from progenitor cells, specific engineering tools, such as fluorescent probes and siRNA, cannot be naturally synthesized in parental cells 74. Cell engineering strategies limit the clinical potential of natural exosomes for *in vivo* imaging and gene therapy. Consequently, exosomes should be designed within the extracellular environment to integrate the diagnosis and treatment of nanoscale vesicles based on the methods of incubation with cargos and membrane permeabilizer, extrusion, electroporation, sonication, freeze and thaw cycles, liposome-based transfection, and click chemistry 75.

**Figure 3:**
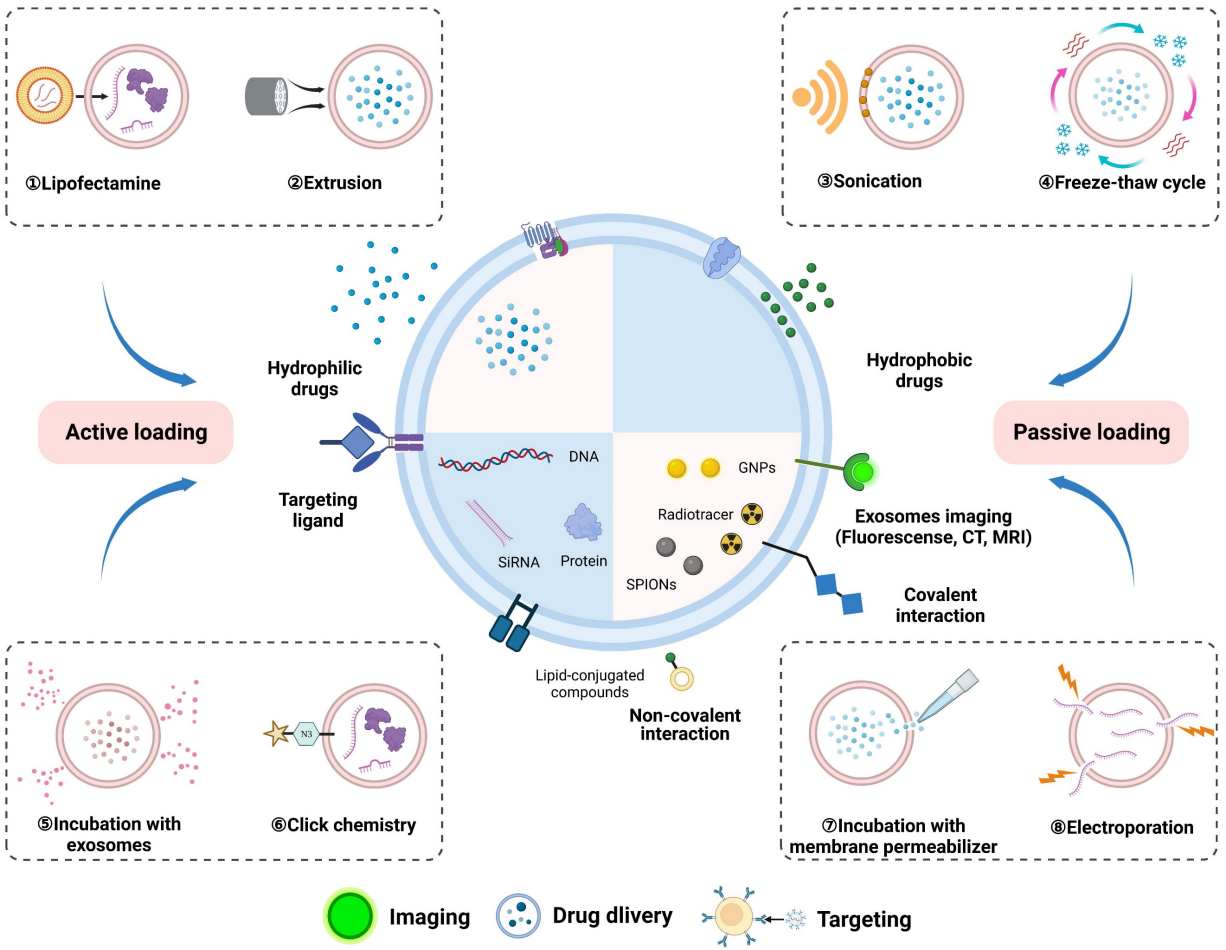
Loading exogenous cargos into exosomes *via* physical and biochemical approaches, including incubation of exosomes with cargos and membrane permeabilizer, extrusion, electroporation, sonication, freeze and thaw cycles, liposome-based transfection, and click chemistry. Exosomes produced by direct functionalization were used for theranostics, such as biological imaging, drug delivery, and targeting cells. Figures created with Biorender.com.

These methods for the direct functionalization of exosomes are as follows (**Figure 3**): (1) Incubation with exosomes involves the binding of hydrophobic cargo to the bilipid layer of the exosomes. Typically, macrophage-derived exosomes were co-incubated with the hydrophobic drug curcumin to produce curcumin-containing exosomes that targeted the cerebral ischemia-reperfusion region 76. (2) Surfactant saponin is usually used as membrane permeabilizer to form a complex with cholesterol on the exosomes membrane to increase the incorporation of external drugs. Exosomes loaded with catalase using saponin permeabilization significantly increased the neuroprotective effects compared with those of the control group, whereas incubation at room temperature without saponin showed no difference 77. (3) To integrate the exosomes membrane, serial extrusion using a lipid extruder is required. Khongkow et al. mixed exosomes and gold nanoparticles (GNPs) in PBS and used a mini extruder to serially extrude them through 400 nm, 200 nm, and 100 nm polycarbonate porous membranes, obtaining brain-targeted exosomes coated with GNPs 78. (4) Electroporation is another method that uses high voltage generated by an electric field to create temporary pores in the exosomes membrane, which is particularly advantageous for siRNA loading. Huang et al. used electroporation to load CTGF gene inhibitors into MSCs-derived exosomes to attenuate spinal cord injury 79. (5) Sonication-produced mechanical shear forces result in the loss of exosomes membrane integrity. The ultrasound-mediated direct functionalization of exosomes achieved a high loading efficiency and sustained drug release, surpassing the quantitative effects of electroporation and co-incubation 80. After sonication treatment of exosomes and berberine, the drug loading reached 17.13%±1.64%, and the cumulative release amount of loaded sample reached 71.44±2.86% within 48 h, thus alleviating inflammation and cell apoptosis caused by SCI 81. (6) Freeze and thaw cycles encompass three subsequent cycles: incubation at room temperature, rapid freezing at -80 ℃ or in liquid nitrogen, and thawing at room temperature. The phospholipid bilayer of exosomes can bind to liposomes enabling the load of molecules with a high capacity through freeze-thawing methods. For verification, conductive polypyrrole nanoparticles were encapsulated in liposomes and fused with MSCs-derived exosomes to facilitate nerve regeneration *via* electrical stimulation 82. (7) Liposome-based transfection involves the fusion of liposomes carrying exogenous genes with exosomes 83. (8) Proteins on exosomes membrane have abundant amino and alkyl groups that allow for their modification with biomacromolecules through covalent interactions. Click chemistry exhibits the attributes of rapidity, high efficiency and enhanced control over the conjugation site. Hosseini Shamili et al. modified exosomes with carboxylic acid-functionalized aptamers using EDC/NHS chemistry for application in neural myelination 84.

While these engineering techniques help exosomes to load exogenous cargoes for imaging, drug delivery, and targeting, their disadvantages cannot be ignored in the process of application, which include exosomes aggregation, loss of membrane integrity, toxicity, hemolytic activity, unspecific release of drugs 75. These challenges highlight the need for careful consideration and appropriate strategies to optimize the use of exosomes as theranostic vehicles.

## Systematic and local delivery of exosomes *in vivo*

**Figure 4:**
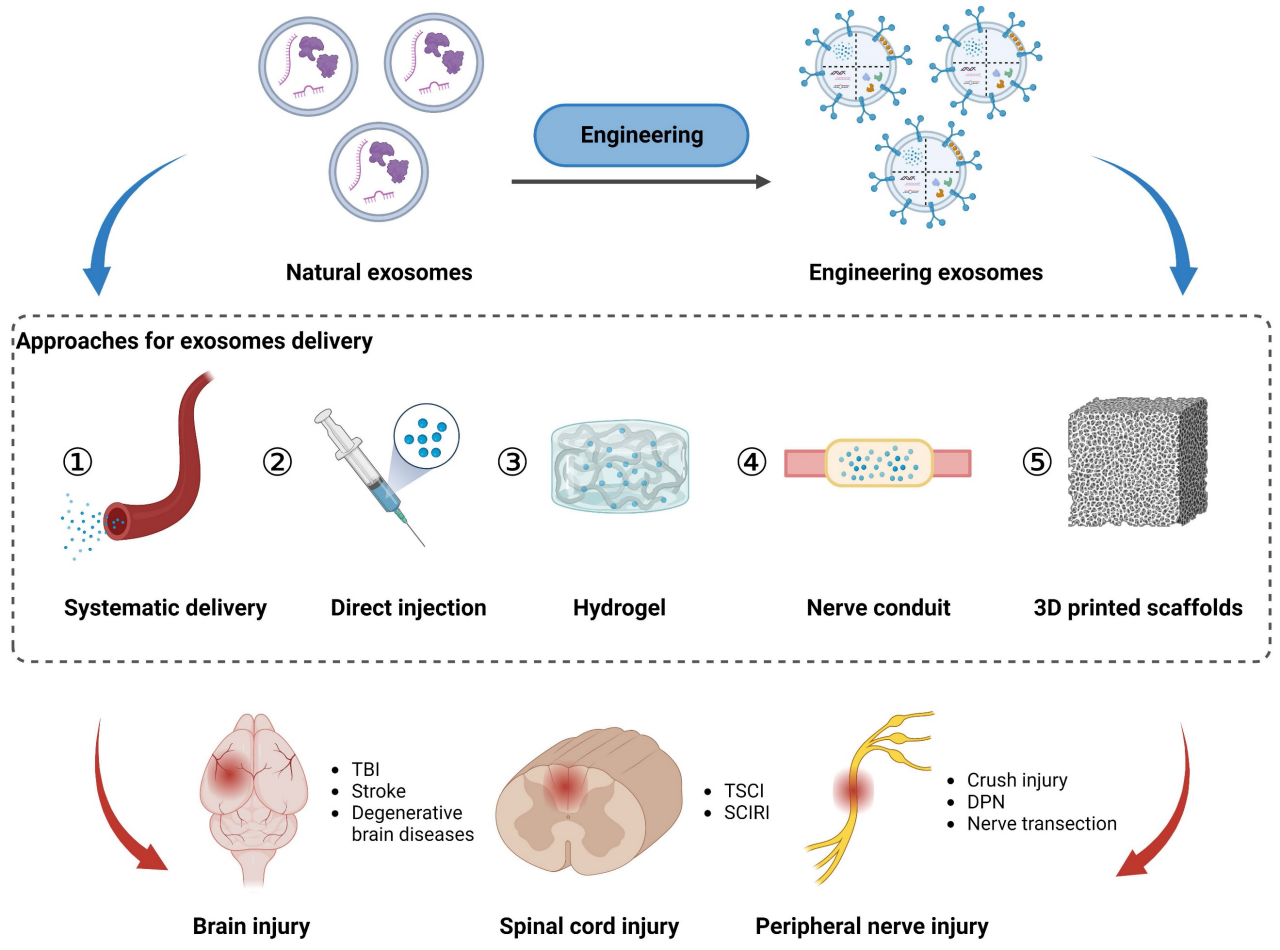
Approaches for exosomes delivery to the injured sites in peripheral nerve, spinal cord, and brain *via* local application (direct injection, hydrogel, nerve conduit, and 3D printed scaffolds) and systematic delivery. Figures created with Biorender.com.

As non-living nanovesicles, exosomes can be integrated into multiple biomaterials without nutritional support. Considering the structural and functional properties of the PNS and CNS, tailored delivery strategies are necessary to achieve safe, targeted, and sustained therapeutic effects of exosomes. In this context, this section discusses the importance of optimized delivery routes, including intravascular injection, hydrogels, nerve conduits, and 3D printed scaffolds, to facilitate the efficient transportation of exosomes to specific sites within the nervous system (**Figure 4**).

### Systematic delivery of exosomes *via* intravascular injection

Systematic delivery of exosomes using a syringe and needle after mixing them with low- concentration PBS or normal saline is the most therapeutic approach for treating nerve injury *via* intravascular injection. Several researches have demonstrated the efficacy of injecting exosomes into animal models of spinal cord injury *via* the tail vein. For instance, Peng et al. injected exosomes derived from the microglial cells into mouse model of traumatic spinal cord injury 85. They observed that the exosomes began to perform their functions as early as 7 days after surgery, along with significant proliferation of endothelial cells, recovery of neurological function, and a reduction of reactive oxygen species (ROS) levels in the injured area. *In vitro*, endothelial cells can rapidly internalize microglial-derived exosomes within 12 h, reducing ROS production in H_2_O_2_-induced bEnd.3 cells and promoting the proliferation, migration, and tube formation by activating the keap1/Nrf2/HO-1 signaling pathway. Fan et al. reported a similar administration route for delivering exosomes derived from the BMSCs into a rat model of spinal cord injury and observed therapeutic effects after consecutive tail vein injections for 7 days 86. These experimental results indicate that exosomes can serve as drug nanocarriers, cross biological barriers, and target damaged tissues through systematic applications. However, it is unreasonable to inject exosomes into blood vessels at high concentrations and high frequencies, because the delivery route may cause cytotoxicity of exosomes and local infection at the injection site 87. Moreover, a large number of exosomes tend to accumulate in the liver, spleen, and lungs through intravascular injection, where they undergo metabolic clearance, thereby limiting the number of exosomes transported to the injured sites 88. Therefore, seeking the localized maintenance and diffusion of exosomes as a substitute for the injection-based route is a favorable strategy for nerve injury repair.

### Hydrogel-mediated sustained release of exosomes

Hydrogels are widely used in regenerative medicine owing to their ability to mimic the extracellular matrix and provide a favorable environment. Encapsulating exosomes within hydrogels offers a way to address the limitations of both exosomes and hydrogels. The incorporation of exosomes into the hydrogel network allows for extended release and enhanced utilization of the exosomes, while the hydrogel acquires the bioactivity from the parent cells. Li et al. combined exosomes from the MSCs with the adhesive peptide PPFLMLLKGSTR-modified hyaluronic acid (HA) hydrogel (Exo-pGel) to repair spinal cord injury lasting more than 4 weeks in rodents, and they observed that more than 90% of the exosomes exhibited sustained release from the 3D pGel for approximately 11 days *in vitro* 89.

These results may be attributed to the viscosity of HA, which facilitates the slow release of exosomes, and their inherent reparative effects at the nerve injury sites. However, in view of the rapid degradation and slow solidification of HA *in vivo*, HA hydrogel is unsuitable for engineering applications in severe spinal cord injury. Luo et al. proposed a solution to address the shortcomings of HA 90. They constructed a photocrosslinkable hydrogel composed of a specific ratio of GelMA, HA-NB, and photo-initiator (LAP) (GelMA/HA-NB), along with exosomes derived from macrophages, for spinal cord injury recovery. After 28 days of soaking in PBS, the hydrogel released 70% of the accumulated exosomes. Moreover, its injection into the spinal cord injured area significantly promoted blood vessel regeneration and functional recovery, which was related to the presence of OTULIN protein in exosomes, activating the Wnt/β-catenin signaling pathway. On the other hand, Han et al. mixed exosomes from 3D-cultured MSCs with GelMA solution and photo-initiator to synthesize a blend solution for exploring the effect of microneedle array patches on spinal cord injury, which was then cast into a nanowell array mold to form GelMA-MN@3D-Exo square patches 91. The GelMA-MN@3D-Exo patches were evaluated for spinal cord injury that demonstrated great reduction of the inflammatory response, played a neuroprotective role, and reversed the glial/fibrotic scar formation resulting from the excessive aggregation of astrocytes.

### Integration of exosomes with other biomaterials

Parallel to hydrogels, various biomaterials have been developed in combination with exosomes for nerve repair. For sciatic nerve dissection injuries, many studies have demonstrated the necessity of bridging between the proximal and distal axons using nerve conduits 92. Currently, chitosan-based biodegradable nerve conduits are used in clinical treatment, and studies have shown that combining chitosan conduits with exosomes holds great therapeutic potential for peripheral nerve regeneration. Rao et al. successfully repaired sciatic nerve injury by incorporating exosomes derived from gingival MSCs into chitosan conduits. However, exogenous exosomes may leak out of the conduit and rapidly degrade *in vivo* 93. To effectively and sustainably harness the therapeutic potential of exosomes, a dopamine-modified chitosan conduit loaded with BMSCs-derived exosomes can stably release exosomes to promote the functional recovery of damaged sciatic nerve 94. Additionally, engineered exosomes enriched with neurotrophic factor gene have been developed, which can be stably released for at least 2 weeks when mixed with an alginate hydrogel 95. Rat ADMSCs were successfully loaded with the NT-3 gene sequence through virus-mediated transfection, resulting in exosomes derived from modified MSCs exhibiting high expression of NT-3 mRNA. These NT-3-overexpressed exosomes, combined with alginate hydrogel, were injected into the silicone conduit to assess their therapeutic effects on a 10 mm sciatic nerve defect *in vivo*. The results showed significant nerve regeneration and functional recovery of the gastrocnemius muscles after 8 weeks. However, the silicone conduit chosen for this study was not degradable and had a high risk of infection and nerve compression in the later stages. With the development of 3D bioprinting in regenerative medicine, 3D printed scaffolds leverage their excellent porosity and interpore channel structure to provide an encapsulation space for exosomes, enabling their controlled release, while also supporting the adhesion of neighboring cells and tissues to the scaffold 96. We have listed recent reports on exosome-incorporated biomaterials in **Table 3** and discuss them extensively in the section on the application of exosomes in peripheral and central nerve injuries. Although the therapeutic potential of exosomes is undeniable, further exploration is required for their clinical application in nerve repair to achieve long-term and localized treatment at the injured sites.

## Application of exosomes in peripheral and central nerve injuries

To date, numerous studies have reported the theranostic potential of exosomes in the PNS and CNS. Notably, the exosomes used in these studies were either loaded with different exogenous cargos or exerted their effects on recipient cells *via* different delivery routes. In this context, the review provides an overview of the applications of exosomes in neuroimaging and emphasizes their comprehensive applications in peripheral nerve, traumatic spinal cord, and traumatic brain injuries.

### Neuroimaging

**Figure 5:**
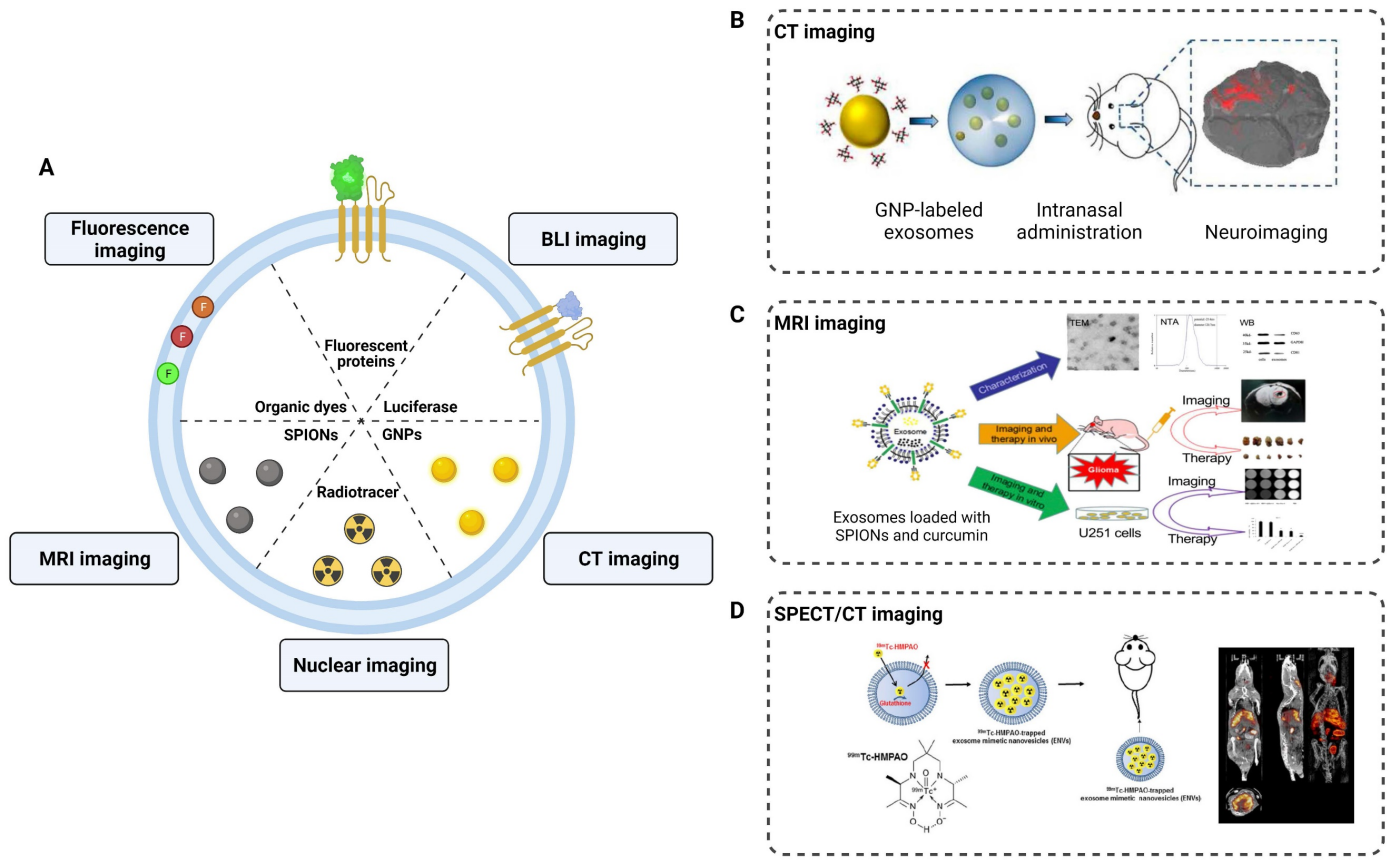
** Application of exosomes in imaging and tracking. (A)** Labeling strategies of exosomes as natural carriers for diagnosis and treatment. **(B)** CT imaging of MSCs-derived exosomes using gold nanoparticles as labeling agents. Adapted with permission from 114, copyright 2017, American Chemical Society. **(C)** Exosomes were loaded with SPIONs and curcumin by electroporation for MRI imaging and therapy of glioma. Adapted with permission from 113, copyright 2018, Elsevier. **(D)** ^99m^Tc-HMPAO was chosen as a proper radiolabeling of exosome-mimetic nanovesicles (ENVS) to show high brain uptake by SPECT/CT imaging. Adapted with permission from 115, copyright 2015 Springer Nature.

Engineering modifications of exosomes have expanded, changed, and enhanced their therapeutic potential and facilitated neuroimaging. To track exosomes *in vivo*, various strategies have been developed to effectively label exosomes (**Figure 5**). Fluorescence imaging, photoacoustic imaging (PAI), CT imaging, MRI imaging, and nuclear imaging (SPECT, PET) are used to non-invasively monitor the absorption, distribution, metabolism and excretion of exosomes. These techniques provide substantial support in elucidating the role of exosomes in the diagnosis and treatment of neurological diseases.

Fluorescence imaging is a commonly employed technique in neuroimaging studies of exosomes. By labeling the surface of exosomes with fluorescent probes, researchers can observe and track the distribution of exosomes in injured nerves. For instance, Bucan et al. used PKH26-labeled exosomes to track the uptake of exosomes by the Schwann cells *in vitro* and explored their distribution in injured sciatic nerves 111. However, traditional fluorescence imaging has limitations in terms of tissue penetration depth and fluorescence decay, which hinder the tracking of exosomes in the central nervous system. The blood-brain barrier (BBB) is the primary obstacle for most contrast agents and drugs to enter the brain. Studies have demonstrated that exosomes can traverse the BBB, offering significant prospects for the diagnosis and treatment of CNS diseases. Perets et al. employed GNPs-labeled BMSCs-derived exosomes and performed CT imaging to elucidate their migration and homing patterns in various brain diseases (stroke, autism, Parkinson's and Alzheimer's disease) 112. The results indicated that the BMSCs-derived exosomes were selectively taken up and accumulated by the neuronal cells in the pathological areas, whereas only diffusive migration with subsequent clearance within 24 h was observed in the control group. Compared to CT imaging, MRI provides higher tissue resolution and avoids radiation exposure in the nervous system. Jia et al. loaded superparamagnetic iron oxide nanoparticles (SPIONs) into exosomes conjugated with myelin basic protein-1 targeting peptide and encapsulated curcumin within the exosomes, achieving targeted MRI imaging and therapy for brain gliomas 113. In addition, 3D images and the distribution of radionuclide-labeled exosomes in organs can be obtained through SPECT or PET detection. When combined with CT or MRI in hybrid devices, accurate localization of labeled exosomes can be achieved for diagnostic purposes.

Engineering modifications of exosomes provide assurance for the development of neuroimaging techniques. In the future, nuclear imaging, which enables the accurate localization of lesions, along with radiation-free MRI imaging combined with the targeting properties of exosomes, can provide high-resolution structural information for the diagnosis of PNS and CNS diseases.

### Peripheral nerve injury

**Figure 6:**
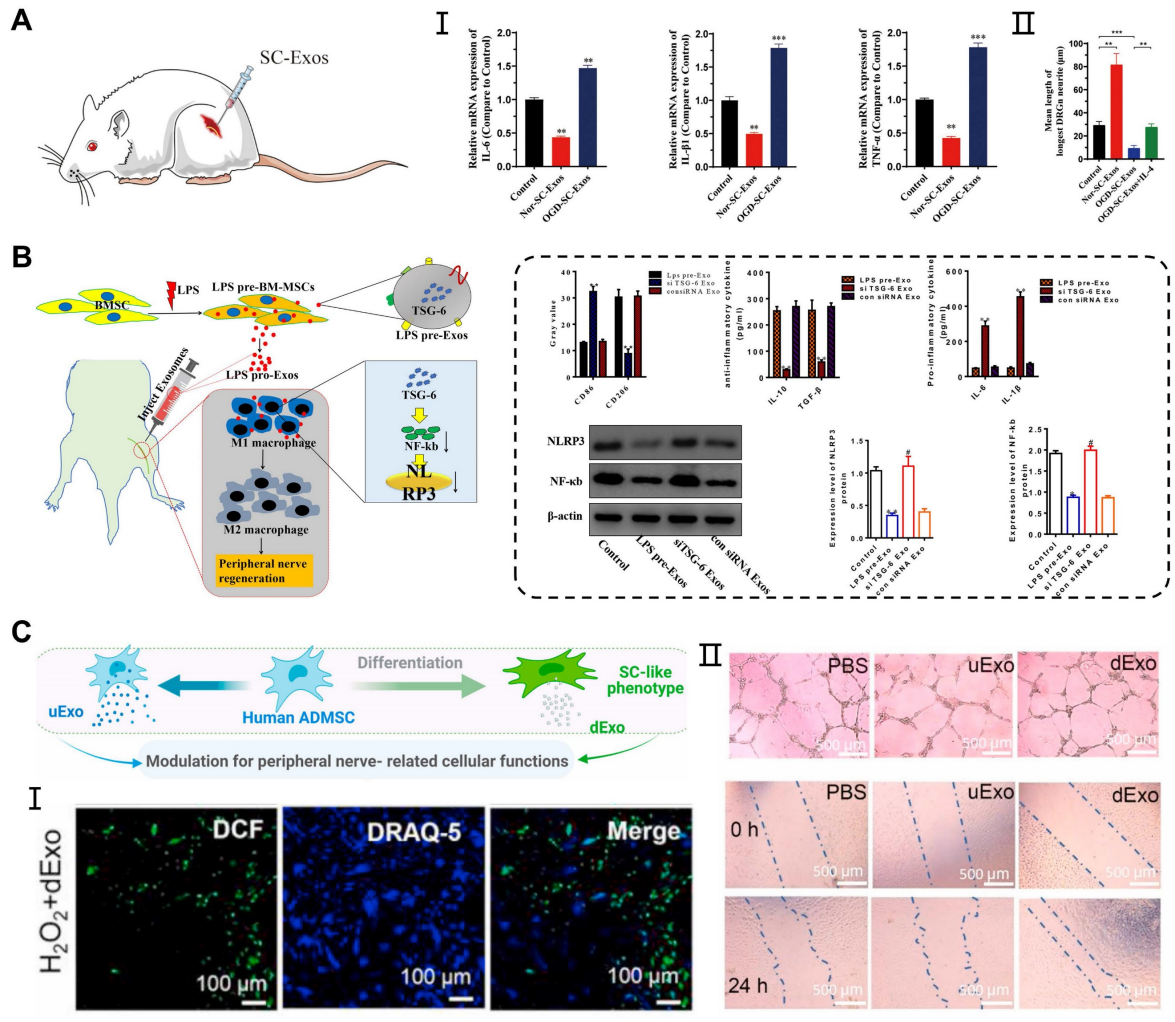
** Application of exosomes in peripheral nerve injury. (A)** OGD-SC-Exos induced the mRNA expression of M1 macrophage markers (IL-6, IL-1β, and TNF-α) *in vivo* and inhibited the neurites elongation of DRG neurons *in vitro*. Adapted with permission from 117, copyright 2023, Elsevier. **(B)** Exosomes derived from LPS-preconditioned MSCs promoted the shift of pro-inflammation macrophage into M2 macrophage phenotype through TSG-6 carried by exosomes inhibiting NF-κB/NLRP3 signaling pathway. Adapted with permission from 120, copyright 2022, Elsevier. **(C)** Exosomes derived from differentiated hADMSCs with the SCs phenotype attenuated the intracellular oxidative stress of primary SCs and enhanced the tube formation, migration of HUVECs. Adapted with permission from 122, copyright 2021, Elsevier.

Peripheral nerve injury (PNI) is a complex and prevalent neurological disorder that affects the nerve fibers, supporting cells (Schwann cells), and blood vessels in the PNS. The Schwann cells are involved in the formation of the peripheral nerve myelin sheath, and studies have revealed that exosomes can act directly on the Schwann cells, facilitating peripheral nerve regeneration. In previous study, ADMSCs-derived exosomes promoted the proliferation, migration, myelination, and secretion of SCs, while also inhibiting cell autophagy 63, 116. Moreover, exosomes derived from the BMSCs combined with PDA-modified chitosan conduit significantly increased the number and diameter of the nerve fibers. This combination promoted myelination by influencing the proliferation, secretion, and reprogramming of the Schwann cells 94. Interestingly, exosomes secreted by the Schwann cells themselves also contributed to nerve repair. Direct injection of exosomes derived from normoxia-conditioned SCs has been found to promote myelination of the sciatic nerve. However, *in vitro* experiments have shown that exosomes originating from oxygen-glucose-deprivation-condition SCs (OGD-SCs-Exos) enhanced M1 polarization, thereby limiting axon regeneration and functional recovery (**Figure 6A**) 117.

During Wallerian degeneration in peripheral nerve injury, macrophages and other immune cells are recruited to the injured sites to promote the clearance of myelin and axonal debris 118. Additionally, the reconstruction of the vascular network can provide an appropriate microenvironment for the regeneration and functional recovery of peripheral nerves 119. Exosomes not only act on the Schwann cells, but also participate in neuroinflammatory responses, oxidative stress, and angiogenesis, contributing to peripheral nerve repair. Experimental studies have shown that local injection of exosomes derived from MSCs pretreated with LPS in a sciatic nerve injury model can induce the transformation of M1 macrophages into M2 polarization, thereby enhancing functional recovery, axon regeneration, and remyelination. RT-qPCR and western blot analysis showed that pretreated exosomes containing TSG-6 contributed to the downregulation of the NF-κB/NLRP3 signaling pathway 120 (**Figure 6B**). On the other hand, exosomes derived from differentiated hADMSCs with the SCs phenotype (dExo) significantly increased angiogenesis and reduced oxidative stress in peripheral nerve regeneration, which was attributed to several upregulated miRNAs within dExo 121 (**Figure 6C**). In peripheral nerve injuries, exosome-mediated intercellular communication between neurons and adjacent cells plays an important role in promoting nerve repair. By surface modification and drug loading into exosomes, we can achieve targeted effects of exosomes on peripheral nerve injury through systematic delivery.

### Traumatic spinal cord injury

**Figure 7:**
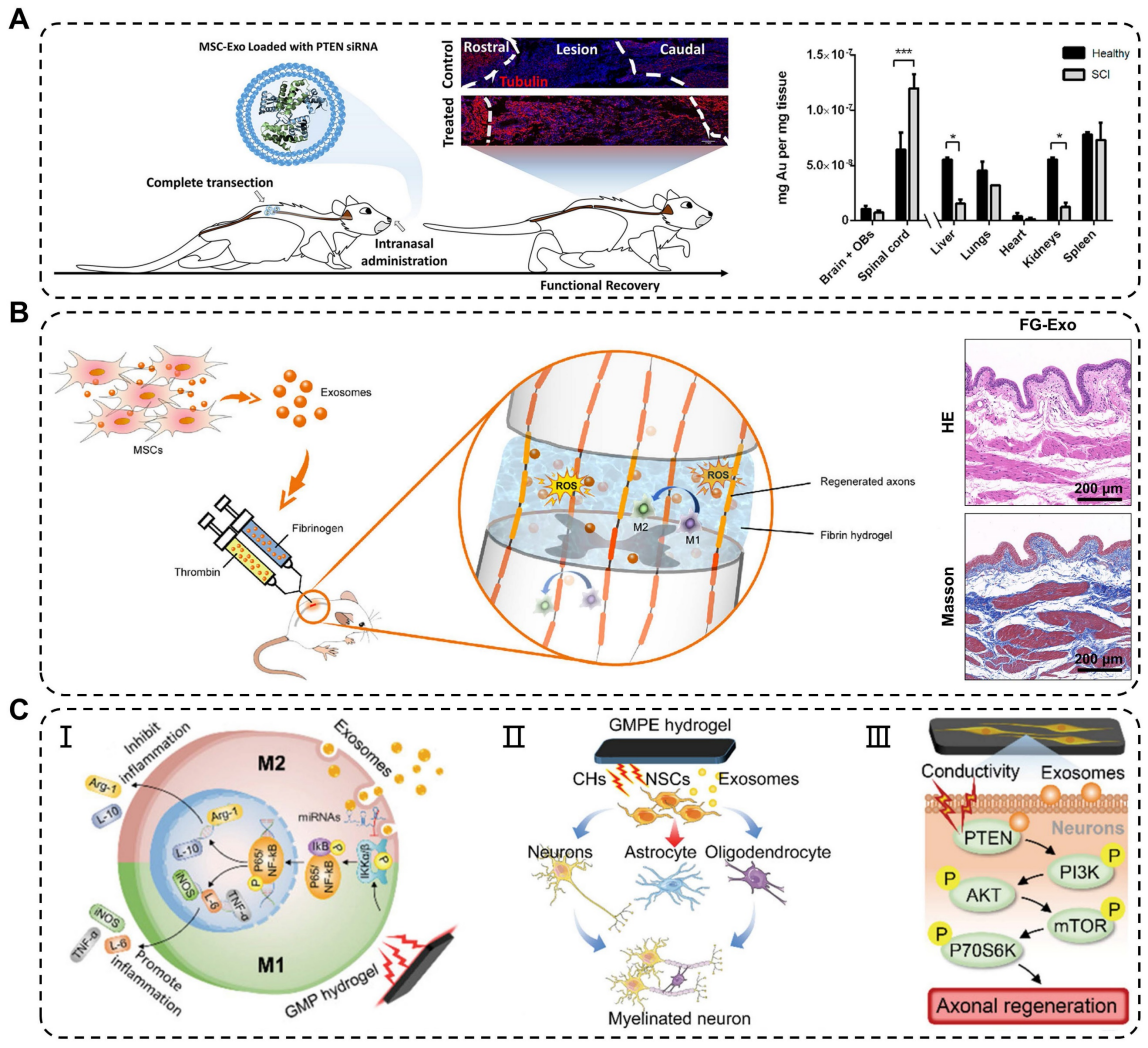
** Application of exosomes in traumatic spinal cord injury. (A)** Intranasal delivery of MSCs-derived exosomes crossed the biological barrier to treat spinal cord injury. Quantitative analysis of gold nanoparticle-labeled exosomes from the CNS and organs in healthy and injured rats. Adapted with permission from 127, copyright 2019, American Chemical Society. **(B)** Reduction in oxidative damage, improvement in M2 macrophages polarization and bladder tissues after fibrin gel-exosomes treatment. Adapted with permission from 102, copyright 2021, Elsevier**. (C)** Mechanisms of exosomes in GMPE hydrogels modulating inflammatory response and axon growth. Adapted with permission from 105, copyright 2022, Wiley-VCH GmbH.

Traumatic spinal cord injury (TSCI) commonly results from in traffic or recreational accidents and often leads to severe neurological dysfunction in the absence of effective treatment 123. In the past, high-dose methylprednisolone pulse therapy was clinically used to treat acute TSCI; however, its effectiveness was limited by serious side effects 124. The novel treatment strategy showed that exosomes can be delivered to the spinal cord through intravenous injection, nasal administration, and local application, thereby circumventing the immunogenicity and tumorigenicity of stem cells transplantation 125. Studies have shown that administration of MSC-derived exosomes *via* tail vein injection into TSCI mice results in significant injury repair, similar to the restorative effects of MSCs transplantation, but without the accompanying drawbacks associated with cell transplantation 126. Additionally, Guo et al. found that exosomes carrying siRNA targeting the phosphatase and tensin homolog (PTEN) reduced its expression in the spinal cord injury area following intranasal delivery 127. The engineered exosomes significantly promoted axon growth and angiogenesis while reducing the proliferation of microglia and astrocytes. However, some exosomes are metabolized by other organs in the bloodstream, thereby limiting their arrival at the injured site and impeding their therapeutic effects (**Figure 7A**). Studies have shown that early local administration of exosomes can regulate acute-phase inflammation; however, direct and systemic delivery of exosomes has certain limitations.

To overcome these limitations, engineering approaches have been developed to combine exosomes with biomaterials, specifically hydrogels, to achieve effective and sustained delivery of exosomes. In one study, Mu et al. reported fibrin gel encapsulation of hUCMSCs-derived exosomes (FG-Exo) for emergency treatment of acute spinal cord injury in rats 102. The results showed that FG-Exo effectively alleviated oxidative stress and inflammatory response in the spinal cord-injured microenvironment, promoted nerve tissue regeneration, and significantly improved urinary system function (**Figure 7B**). In another study, Li et al. modified HA hydrogel with laminin-derived peptide (PPFLMLLKGSTR) to create highly adhesive hydrogel (pGel) that retained human placental MSCs-derived exosomes (Exo-pGel) in a severe SCI model 89. However, natural hydrogels have limited mechanical strength and unstable structures, which restrict their long-term release of exosomes. Synthetic hydrogels, such as GelMA and PLGA hydrogels, are composite biomaterials that offer a balance between biocompatibility and mechanical properties required for spinal cord application. Using 3D GelMA as a delivery system for exosomes (GelMA-Exo) can fill the spinal cord cavity and achieve sustained release. *In vitro*, GelMA-Exo promoted the survival and neural differentiation of NSCs, reduced glial scar formation, increased axon growth, and effectively promoted the recovery of neurological functions in SCI 103. FAN et al. developed a dual-network electroconductive hydrogel (GMP) consisting of photocrosslinking GelMA hydrogel and polypyrrole (PPy) hydrogel. MSCs-derived exosomes were incorporated into the GMP network through reversible non-covalent binding to form the GelMA/PPy/Exosomes hydrogel (GMPE) 105. The application of the GMPE hydrogel to bridge the transected spinal cord resulted in the partial restoration of endogenous electrical signal transmission. Moreover, exosomes released from the GMPE hydrogel can last up to 14 days because the hydrogel has excellent biological adhesion. Implantation of the GMPE hydrogel into the injured spinal cord regulated the polarization of microglia from M1 to M2 through the NF-κB pathway. It also enhanced differentiation of NSCs into neurons and oligodendrocytes and activated the PTEN/PI3K/AKT/mTOR pathway to promote axon growth, synergistically facilitating the recovery of motor function at an early stage of SCI (**Figure 7C**).

Besides the aforementioned biomaterials, self-assembling peptides (SAPs) have been used to repair spinal cord injury 128. Therefore, the strategies of biomaterials for exosomes loading provide promising guidance for clinical practice in the treatment of CNS diseases.

### Traumatic brain injury

**Figure 8:**
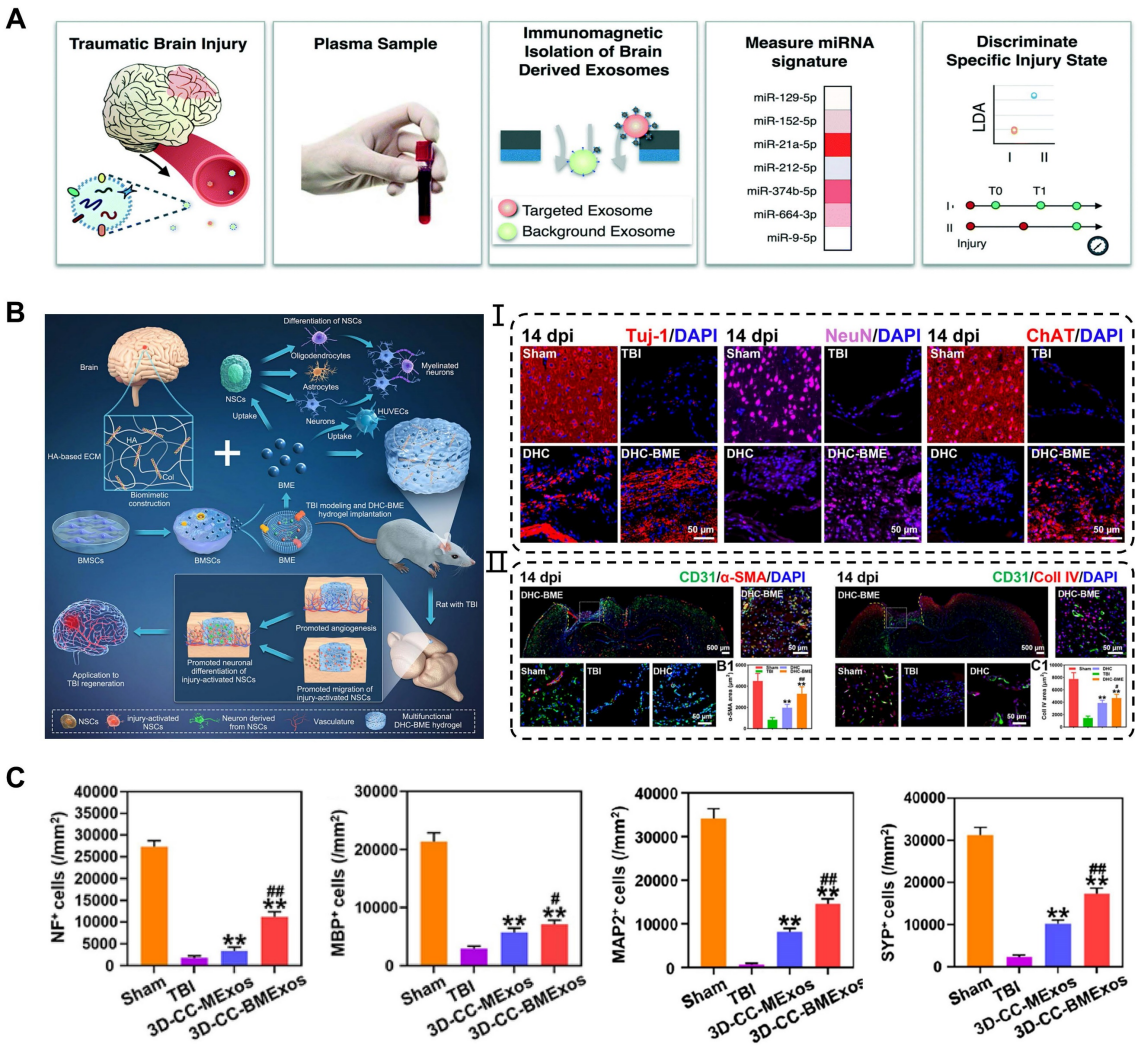
** Application of exosomes in traumatic brain injury. (A)** Machine learning-based TBI diagnostics to analyze brain-derived exosomes using immunomagnetic isolation. Adapted with permission from 130, copyright 2018, Royal Society of Chemistry. **(B)** Exosomes encapsulated in hyaluronan-based hydrogel promoted TBI repair by NSCs differentiation, neurogenesis, and angiogenesis. Adapted with permission from 107, copyright 2023, Elsevier. **(C)** 3D-CC-BMExo increased the number of positive cells for nerve fibers, synaptic connections, and myelin regeneration at implanted site. Adapted with permission from 50, copyright 2022, Oxford University Press.

Traumatic brain injury (TBI) is caused by structural damage and/or functional impairment of brain tissue due to external trauma, and is the leading cause of morbidity and mortality among young people worldwide 129. Severe TBI can be diagnosed on the basis of clinical presentation and imaging observations. However, the existing molecular and imaging examinations for chronic TBI have certain limitations. Hemphill et al. developed a nanofluidic immunomagnetic technique to purify exosomes and analyze their miRNAs 130. The analysis was performed using chips and machine learning, with a set of biomarkers as indicators for TBI grading and diagnosis (**Figure 8A**). In the experiments, the technique achieved a correct recognition rate of 99% in both traumatic and sham-operated mice. The role of natural exosomes is limited in the treatment of brain disease. Increasing evidence suggests that appropriate engineering methods can enhance the therapeutic effects of exosomes in traumatic brain injury with neural damage and functional impairments. Wang et al. investigated the effects of genetically modified exosomes on cell apoptosis and neural function following traumatic brain injury 131. They transfected plasmids carrying Bcl-2 cDNA and Bax shRNA into exosomes derived from astrocytes and then injected functional exosomes into the ventricle after 1 h of surgery. Following 48 h of exosomes treatment, the Mcl-1, XIAP, and Survivin protein levels in the brain were attenuated and the release of cytochrome c from the mitochondria to the cytoplasm was reduced. Moreover, the engineered exosomes mitigated the damage to miniature excitatory postsynaptic currents (mEPSCs) and long-term potentiation (LTP) in the hippocampus of TBI mice, leading to improvement in motor and cognitive behavior.

Although exosomes can be administered intravenously or injected directly for TBI, the application of sustained-release and defect-filling biomaterials optimized their delivery route. Liu et al. combined BMSCs-derived exosomes with HA-collagen hydrogel to simulate the brain matrix and achieved a stable release of exosomes 107. These results revealed that the hydrogel encapsulated exosomes induced angiogenesis and neurogenesis at the injured site, consequently promoting neural function recovery after TBI (**Figure 8B**). Recently, many researchers have successfully integrated bioscaffolds and exosomes using 3D printing technology to attain the desired mechanical properties and biocompatibility of biomaterials for TBI treatment. Liu et al. reported that combining 3D printed collagen/chitosan scaffolds with BDNF-pretreated hUCMSCs-derived exosomes (3D-CC-BMExos) enhanced the reparative effects of exosomes on TBI 50. The bioscaffold exhibited promising biocompatibility and degradation properties, and the incorporation of 3D-CC-BMExo further promoted neural network remodeling by improving nerve fibers, synaptic connections, and myelin regeneration at the implanted site (**Figure 8C**). Similarly, the research team treated TBI in a beagle model by combining 3D printed collagen/silk fibroin scaffolds with exosomes obtained from hypoxia-pretreated hUCMSCs (3D-CSHMExos) and found that 3D-CSHMExos significantly promoted neural regeneration and angiogenesis. Furthermore, 3D-CSHMExos further inhibited neuronal apoptosis and pro-inflammatory cytokines (TNF-α and IL-6) while increasing the expression of anti-inflammatory cytokine (IL-10), ultimately facilitating motor function recovery after TBI 109. In this review, we provided a concise overview of the optimized application of exosomes in TBI. However, current research is still confined to the animal models, necessitating extensive investigation for clinical applications.

## Conclusion and Perspectives

Neurological disorders can arise from various causes, such as tumors, autoimmunity, and genetic factors, aside from direct damage to the axons and cell bodies of the peripheral and central nerves. Traditional therapeutic strategies only provide symptomatic relief on a macroscopic level, without fully restoring neural structure or removing risk factors. Exosomes, with nanomembrane structures, have the ability to effortlessly cross the BBB, blood-spinal cord barrier, and blood-nerve barrier while maintaining their biological activity. These features present a promising therapeutic and diagnostic strategy for neurological diseases, for which extensive research has been conducted on the mechanisms, delivery pathways, and production improvement. Exosomes carry a variety of molecules on their surface and interior, including integrins, ceramides, MHC, proteins, RNA, and DNA; however, not all properties can be fully utilized in the application process. Thus, exosomes need to be functionalized for specific purposes, such as targeting, bioimaging, and drug loading. Advancement in cell engineering, genetic engineering, and biomaterials technology have provided technical support for these specific applications of exosomes. Enhancing our understanding of the biogenesis of exosomes by regulating parental cell culture parameters and utilizing various bioreactors have improved exosomes production for basic research; however, current isolation techniques greatly restricted their clinical potential.

The focus of exosomes research has progressively shifted towards clinical applications for the treatment of various diseases. Chu et al. utilized exosomes derived from MSCs for nebulized therapy in seven COVID-19 patients, contributing to improving the absorption of lung lesions and reducing hospitalization time for mild cases of COVID-19 132. However, there are still no reports of clinical trials investigating the use of exosomes for nerve injury repair (source: ClinicalTrials.gov). In order to provide researchers with guidance on producing exosomes in accordance with good manufacturing practices, the Minimal Information for Studies of Extracellular Vesicles (MISEV) from the International Society for Extracellular Vesicles (ISEV) has provided extensive information and instructions 133. Nevertheless, there is no detailed explanation of the safety issues related to the surface modification and gene loading of exosomes, and the potential side effects in clinical applications remain unclear. Moreover, the clinical application of exosomes raises ethical and safety concerns owing to the challenges associated with the cell source for exosomes secretion, particularly in the context of allogeneic sources, and the potential contamination of extracted exosomes with other proteins, which has not been fully demonstrated in clinical trials. Even when using MSCs from autologous sources, meeting the high demand for exosomes remains challenging, especially considering the criticality of early treatment for nerve injury. Hence, mechanical stimulation and dynamic culture system are necessary to promote exosomes production for the preparation of therapeutic reagents. However, these parameters must be strictly adjusted to avoid the overexpression of inflammatory factors and non-exosomes vesicle shedding. Various biological repair materials have been widely applied in clinical treatment, ensuring their safety and stability, but further research is needed to explore their combined application with exosomes for the effective treatment of neurological diseases. Despite being undeniably crucial for nerve injury repair, exosomes are relatively novel drug used in the field of tissue engineering. Therefore, further research on the clinical efficacy and production of exosomes is necessary before their safe application in humans.

## Figures and Tables

**Table 1:** List of multiple factors to enhance exosomes bioactivity

Extracellular factors		Exosomes source	Effect on exosomes	Molecular mechanism	References
**Culture conditions**	Low culture density	BMSCs	Increased production of exosomes	N/A	30
	Hypoxia	UCMSCs	Angiogenesis, promoted functional recovery after injury	HIF-1α↑, VEGF↑	32
	3D culture	UCMSCs	Increased production of exosomes and siRNA delivery to neurons	N/A	34
	Mild hypothermia	Microglia	Upregulated the expression of miR-20b-5p, promoted neurite outgrowth and synapse recovery	PTEN↓, PI3K/AKT↑	47
	Hypocapnia	ADMSCs	Promoted cellular activity and regeneration of nerve fibers	N/A	48
**Biochemical factors**	LPS, IFN-γ, TNF-α	BMSCs	Anti-inflammation	NF-κB↓, AKT1/AKT2↑; COX2/PGE2↑	35, 36
	Fe3O4, Iron oxide nanoparticles	BMSCs	Angiogenesis, targeting injured sites	SPRY2↓, PI3K/AKT and ERK1/2↑; N/A	37, 38
	IGF-1	NSCs	Enrichment of miRNA level, anti-inflammation, anti-apoptosis	miR-219a-2-3p↑, YY1↓, NF-κB↓	49
	BDNF	UCMSCs, BMSCs	Remodeled neural networks, inhibited apoptosis	N/A; miR-216a-5p↑	50, 51
	FK506	ADMSCs	Enhanced nerve regeneration, reduced autophagy, enrichment of protein level	N/A; HSPA8 and EEF1A1↑	52, 53
**Mechanical factors**	Hollow fiber bioreactors	UCMSCs	Angiogenesis	TGF-β1 and Smad2/3↑	44
	Vertical-wheel bioreactors	BMSCs, ADMSCs, UCMSCs	Increased production of exosomes	N/A	45
	Low-intensity pulsed ultrasound (LIPUS)	Schwann cells	Enhanced axon elongation, promoted SCs proliferation	PI3K/AKT/FoxO↑	46
**Environmental enrichment**	Youthful systemic milieu	Serum	Promoted OPC differentiation and myelination	miR-219↑	54
	Physical, intellectual and social activities	Immune cells	Promoted CNS myelination, reduced LPS-induced astrogliosis	miR-219↑	55

**Table 2:** Molecular mechanism of miRNA-containing exosomes in nerve repair

microRNA	Cell source	Biological function	Molecular mechanism	Application	References
miR-22-3p	ADMSCs	Enhanced SCs proliferation and migration	PTEN↓, p-AKT/mTOR↑	Peripheral nerve injury	62
miR-26b	ADMSCs	Promoted myelination, reduced autophagy of injured SCs	Kpna2↓	Sciatic nerve crush injury	63
miR-214	MDSCs	Improved SCs function and axon extension	PTEN↓, JAK2/STAT3↑	Sciatic nerve crush injury	24
miR-199a-3p/145-5p	UCMSCs	Promoted PC12 cell differentiation, facilitated spinal cord functional recovery	NGF/TrkA↑	Traumatic spinal cord injury	64
miR-672-5p	M2 microglial	Promoted axon regeneration, reduced neural pyroptosis	AIM2/ASC/caspase-1↓	Traumatic spinal cord injury	65
miR-499a-5p	ADMSCs	Reduced neuronal apoptosis, improved the functional recovery	JNK3/c-jun↓	Spinal cord injury	26
miR-133b	ADMSCs	Promoted expressions of NF, GAP-43, GFAP, and MBP	p-CREB/CREB↑, p-STAT3/STAT3↑	Spinal cord injury	66
miR-125a	BMSCs	Promoted the recovery of locomotor function and M2 polarization, inhibited neuronal apoptosis and inflammatory response	IRF5↓	Spinal cord injury	61
miR-133b	BMSCs	Preserved neurons, promoted axon regeneration, improved the recovery of locomotor function	ERK1/2, STAT3, CREB↑	Spinal cord injury	60
miR-124-3p	BMSCs	Impeded cell apoptosis, enhanced M2 polarization	Ern1↓	Spinal cord ischemia-reperfusion injury	67
miR-124-3p	microglia	Promoted M2 microglial polarization, inhibited neuronal autophagy,	PDE4B/mTOR↓; FIP200↓	Traumatic brain injury	19, 68
miR-21-5p	HT-22 neurons	Inhibited neuronal autophagy	Rab11a↓	Traumatic brain injury	69
miR-873a-5p	Astrocytes	Attenuated microglia-mediated neuroinflammation, improved neurological deficits	NF-κB↓	Traumatic brain injury	18
miR-181b	BMSCs	Reduced apoptosis and neuroinflammation, promoted M2 microglia polarization	IL10/STAT3↑	Traumatic brain injury	70
miR-182-5p	Astrocytes	Inhibited neuroinflammation	Rac1↓	MCAO/R	71
miR-17-92 cluster	BMSCs	Promoted axonal growth, enhanced neuroplasticity and functional recovery	PTEN/mTOR↑; PI3K/AKT/mTOR/GSK-3β↑	Brain injury (traumatic brain injury, MCAO)	72, 73

**Table 3:** Recent studies using exosomes biomaterials for nerve injury repair in animal Models

Biomaterials	Exosomes source	Species	Disease	Biological function	Reference
Chitin conduit	GMSCs	Rat	Sciatic nerve defect	Increased nerve fiber regeneration and myelinization	93
Polydopamine-modified Chitin conduit	BMSCs	Rat	Sciatic nerve defect	Promoted Schwann cells proliferation and nerve regeneration	94
Silicone tube with exosomes-alginate hydrogel	ADMSCs	Rat	Sciatic nerve defect	Promoted nerve regeneration and maintained muscle morphology	95
rGO-GelMA-PCL conduit	BMSCs	Rat	Sciatic nerve defect	Enhanced angiogenesis and axon growth	97
Core-shell silk-fibroin/PLLA nerve conduit	EnSCs	Rat	Sciatic nerve defect	Enhanced axons regeneration, improved functional recovery	98
Hyaluronic acid methacrylate hydrogel	UCMSCs	Rat	Sciatic nerve crush injury	Exosome-loaded soft hydrogel promoted nerve repair and anti-inflammation	99
Alginate scaffold	UCMSCs	Rat	Spinal nerve ligation pain	Antinociceptive, anti-inflammatory, and pro-neurotrophic effects	100
Peptide-modified adhesive hydrogel	Placenta amniotic membrane MSCs	Rat	Spinal cord injury	Promoted spinal cord regeneration and protected urinary tissue by antioxidation and anti-inflammation	89
PPFLMLLKGSTR-modified HA hydrogel	UCMSCs	Rat	Spinal cord injury	Replenished spinal cavity, enhanced angiogenesis and locomotor function recovery	32
Fibrin gel	BMSCs	Mouse	Spinal cord injury	Upregulated neural markers, promoted nerve regeneration and oligodendrogenesis by VGF	101
Fibrin gel	UCMSCs	Rat	Spinal cord injury	Relieved inflammation and oxidative damage, protected urinary function	102
Gelatin methacrylate hydrogel	BMSCs	Rat	Spinal cord injury	Promoted neurogenesis, attenuated glial scars	103
F127-polycitrate-polyethyleneimine hydrogel	ADMSCs	Rat	Spinal cord injury	Suppressed scar formation, reduced inflammatory reaction, promoted remyelination and axonal regeneration.	104
Gelatin methacrylate/ polypyrrole electroconductive hydrogel	BMSCs	Mouse	Spinal cord injury	Anti-inflammation, enhanced neural stem cells differentiation and axonal growth, inhibited astrocyte differentiation	105
GelMA hybrid microneedle array patch	MSCs	Rat	Spinal cord injury	Reduced SCI-induced inflammation and glial scarring	91
Polydopamine-modified hydrogel	Flos sophorae immaturus	Rat	Spinal cord injury	Reduced inflammation and oxidative damage, promoted motor and urinary function	106
Hyaluronan-collagen hydrogel	BMSCs	Rat	Traumatic brain injury	Induced angiogenesis and neurogenesis, promoted axon growth, remyelination, and synapse formation	107
Nano-SDF scaffold	NSCs	Rat	Traumatic brain injury	Decreased oxidative stress, reactive gliosis, and neuroinflammation, increased neurogenesis	108
3D-printed collagen/silk fibroin scaffold	UCMSCs	Canine	Traumatic brain injury	Inhibited nerve cell apoptosis and proinflammatory factors expression, enhanced motor function recovery	109
3D-printed collagen/chitosan scaffold	UCMSCs	Rat	Traumatic brain injury	Recovered neuromotor and cognitive function, improved the regeneration of nerve fibers, synaptic connections, and myelinization	50
Low-temperature 3D-printed collagen/chitosan scaffold	NSCs	Rat	Traumatic brain injury	Improved motor and cognitive functions, recovered damaged nerve tissue	110

## Abbreviations

ADMSCs: adipose-derived mesenchymal stem cells
BMSCs: bone mesenchymal stem cells
BNDF: brain-derived neurotrophic factor
CNS: central nervous system
CTGF: connective tissue growth factor
COVID-19: corona virus disease 2019
DRG: dorsal root ganglion
EnSCs: endometrial stem cells
ESCRT: endosomal sorting complex required for transport
EVs: extracellular vesicles
GelMA: gelatin methacryloyl
GMSCs: gingival mesenchymal stem cells
GNPs: gold nanoparticles
HA: hyaluronic acid
HUVECs: human umbilical vein endothelial cells
HIF-1α: hypoxia-inducible 1-alpha
IGF-1: insulin-like growth factor 1
ISEV: international society for extracellular vesicles
ILVs: intraluminal vesicles
KDR: kinase insert domain receptor
LTP: long-term potentiation
LIPUS: low-intensity pulsed ultrasound
MRI: magnetic resonance imaging
MSCs: mesenchymal stem cells
MCAO: middle cerebral artery occlusion
mEPSCs: miniature excitatory postsynaptic currents
MISEV: minimal information for studies of extracellular vesicles
MVBs: multivesicular bodies
MDSCs: muscle-derived stem cells
NSCs: neural stem cells
OGD/R: oxygen-glucose deprivation and reperfusion
PNS: peripheral nervous system
PNI: peripheral nerve injury
PBS: phosphate buffered solution
PLGA: poly(lactic-co-glycolic acid)
PLLA: poly-L-lactic acid
PET: positron emission tomography
ROS: reactive oxygen species
SCs: Schwann cells
SPECT: single-photon emission computed tomography
SPIONs: superparamagnetic iron oxide nanoparticles
TFF: tangential flow filtration
TBI: traumatic brain injury
TSCI: traumatic spinal cord injury
UCMSCs: umbilical cord mesenchymal stem cells
VEGFA: vascular endothelial growth factor A
